# Quenching Sensitivity of Al-Zn-Mg Alloy after Non-Isothermal Heat Treatment

**DOI:** 10.3390/ma12101595

**Published:** 2019-05-15

**Authors:** Shuai Li, Honggang Dong, Xingxing Wang, Zhongying Liu

**Affiliations:** 1 School of Mechanical Engineering, North China University of Water Resources and Electric Power, Zhengzhou 450045, China; lyctlishuai@163.com (S.L.); paperwxx@126.com (X.W.); liuzhongying87@126.com (Z.L.); 2 School of Materials Science and Engineering, Dalian University of Technology, Dalian 116024, China

**Keywords:** non-isothermal heat treatment, quenching sensitivity, corrosion susceptibility

## Abstract

Type Al-Zn-Mg alloy has a wide ranges of application in vehicles, but corrosion resistance and mechanical properties of this alloy after heat treatment or heat straightening limits its utilization. This paper investigates the effect of quenching condition during non-isothermal heat treatment on corrosion behavior and mechanical properties of Al-Zn-Mg alloy. The corrosion resistance of Al-Zn-Mg alloy decreases after non-isothermal heat treatment and the sample after air quenching has the lowest corrosion resistance, the specimens can get better corrosion resistance when suffered 5 min air cooling followed by water quenching process. The evolution of mechanical properties and corrosion resistance of heat-treated specimens is caused by the modification of distribution of precipitates at grain boundary and the microchemistry of precipitates at grain boundary, precipitate-free zones and the matrix.

## 1. Introduction

Facing severe challenges of energy exhaustion and environmental degradation, the low emission and low energy consumption technologies have become popular topics for researchers, and the lightweight development of transport vehicles, such as railway vehicles, automobiles and airplanes, is one of the effective measures to solve these problems [1].In recent decades, aluminum alloys are widely applied as common structural materials to meet the demand of lightweight due to its advantages in low density, good mechanical properties and corrosion resistance and high recycle utilization rate comparing to traditional steel materials [2,3,4,5,6]. 

7000 series aluminum alloys are often joined through welding in the assembly of structures. The welding of aluminum alloys commonly causes welding distortion due to its special physical parameters. Then welding deformation will give rise to the problem of low dimensional accuracy and high manufacturing cost in the construction process of structural components [7,8,9]. Consequently, it is necessary to regulate welding deformation of welded structural parts during the manufacturing. Welding deformation is affected by several factors, such as local shrinkage, root clearance and symmetry between different structural components [7,8,9].Lots of approaches have been taken to control distortion before or during welding. However, it is difficult to control welding deformation within a certain range of assembly when the distortion is relatively large. Consequently, the heat straightening is always applied to further decrease welding deformation of different structural components. Generally speaking, there are two approaches for correcting post-weld deformation, i.e., mechanical straightening and flame correction (heat straightening) [10]. Mechanical straightening is always employed to correct deformation of small structural parts, while flame correction is suitable for large components such as blocks and subassemblies [10]. Flame correction has been widely used due to its simple facility and flexibility [11,12,13,14,15].

**Figure 1:**
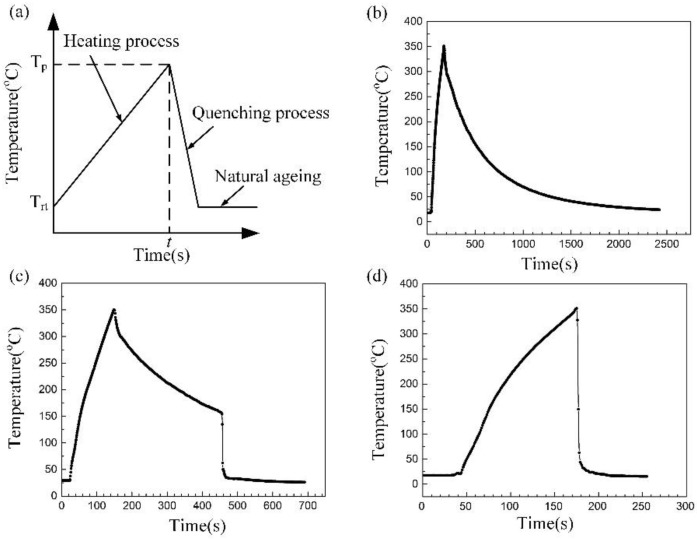
The schematic diagram for heat straightening (**a**) and the thermal cycle curve of the samples (**b**) AQ, (**c**) AQ-5, (**d**) WQ with different quenching conditions.

Flame correction is a procedure in which a limited amount of heat is provided through the torch to localized deformation areas of welded parts by repeating thermal cycles to cause a gradual deformation [11,12,13,14]. Since metallic materials expand when heated and contract when cooled, the metal plate undergoes shrinkage plastic deformation because the expansion is hindered by the relatively cold surrounding area. Then the new shrinkage distortion counteracts the prevenient welding distortion when the temperature is lowered. The thermal straightening is a non-isothermal heat treatment (NIHT) procedure, and primarily includes three steps, namely heating step, quenching process and subsequent natural ageing stage, as shown in Figure 1. The highest temperature of heat straightening for 7000 series alloys usually reaches 350 °C, then dissolution of initial precipitates, diffusion of elements and formation of precipitates occurred simultaneously [16].

Lots of investigations have proposed that the 7000 series alloys often have quenching sensitivity [17,18,19,20,21,22,23,24]. Heterogeneous precipitates can form during quenching cooling and then affect the precipitation process in subsequent artificial ageing [17]. The mechanical properties and corrosion resistance are mainly related to the distribution of precipitates, consequently, the quenching process will lead to the change of mechanical properties and corrosion susceptibility of 7000 series alloys. 

Lots of researches have been carried out on the quenching process after solution treatment [17,18,19,20,21,22,23,24] and few papers focus on the quenching procedure during heat straightening. The precipitates will be subjected to dissolution and re-precipitation during heat straightening. Consequently, it is necessary to study the influence of quenching conditions of NIHT on mechanical properties and corrosion susceptibility of 7000 series alloys. The relevant researchers [25,26] investigated the effect of thermal straightening temperature on mechanical properties and corrosion resistance of 7000 series alloys with the method of direct oxy-acetylene flame heating. The corresponding results are mainly associated with the experience of operators, which is lack of repeatability by using this heating method. Consequently, it is difficult to find out the variation mechanisms of mechanical properties and corrosion susceptibility of 7000 series alloys precisely by this method. So thermal straightening process was carried out through NIHT procedure to simulate the effect of thermal straightening. The aim is to investigate the effect of quenching process on the mechanical properties and corrosion behavior of Al-Zn-Mg alloy during thermal straightening.

## 2. Experimental Procedure

The chemical composition of Al-Zn-Mg alloy with T5 condition extrusion plate is listed in Table 1. The specimens with the sizes of 150 mm × 40 mm × 4 mm were got from Al-Zn-Mg alloy with T5 condition extrusion plate. The thermal cycles for the specimens were conducted in a resistance furnace (KSL-1200X, HeFei KeJing Materials Technology Co., LTD, HeFei, China), a temperature recorder (Yokogawa GP10, Yokogawa Electric Co., Tokyo, Japan) with a K-thermocouple embedded in the geometric center of the specimen was used to record the corresponding heat treatment cycles. 

First, the specimens were heated in the furnace from room temperature to 350 °C, then quenched in different quenching medium immediately. Considering deformation, the structure is commonly heat straightened, then processed with air cooling for 5 min and subsequent mechanical rectification, finally followed by water quenching. We self-defined the acronyms to represent the various samples for convenience of discussion in this paper, the details of acronyms are shown in Table 2 and the corresponding schematic diagram of thermal cycles are displayed in Figure 1. The duration time from room temperature to 350 °C was arranged as around 120 s.

**Figure 2:**
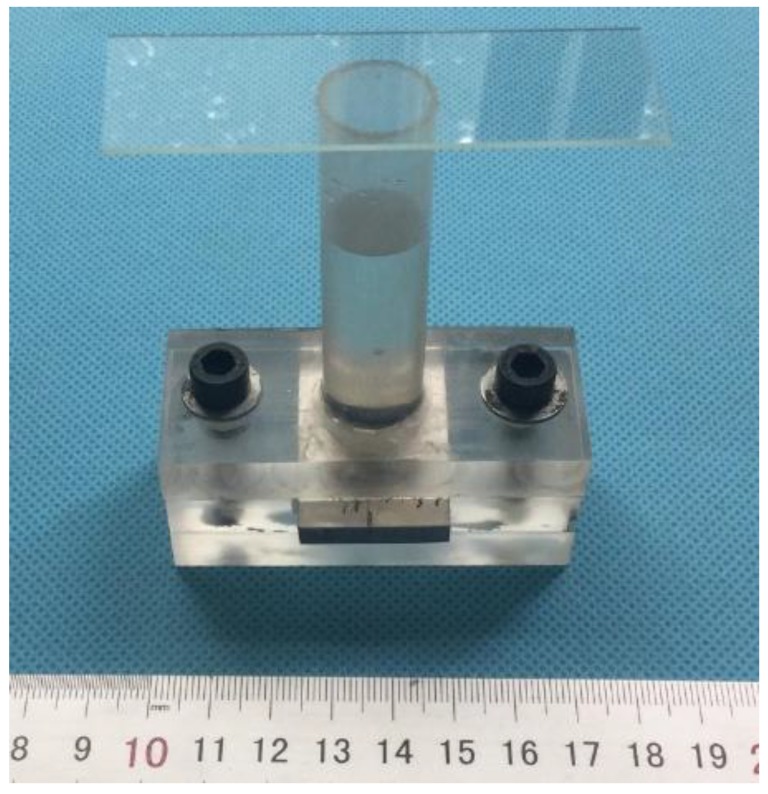
The apparatus for producing corrosion damage.

The intergranular corrosion (IGC) experiment was performed based on criterion ASTM G110-92, and the sizes of specimens are 40 × 25 × 4 mm^3^, which were cut from the specimens after different quenching processes [27]. The IGC test was performed in water bath at 35 ± 2 °C with the solution of 1.0 M NaCl + 0.01 M H_2_O_2_.The intergranular corrosion depth observation was performed through optical microscope (OM). The maximum intergranular corrosion depth was used to analyze the susceptibility of IGC for different heat-treated samples based on the standard of ASTM G110-92 [27].The mixture solution of 4 M NaCl + 0.5 M KNO_3_+ 0.1 M HNO_3_ is prepared for exfoliation corrosion on the basis of standard ASTM G34-01 [28] and the size of samples is 40 × 30 × 4 mm^3^. The exfoliation corrosion rank was evaluated through visual observation. Additionally, the abbreviations used to express different exfoliation corrosion ranks are as follows: Pitting (undermining pitting) P, exfoliation corrosion EA to ED (with EA weak exfoliation corrosion and ED very severe exfoliation corrosion) according to the standard photographs in ASTM G34-01 specification [28]. The average height difference of exfoliation corrosion was obtained based on confocal laser scanning microscope (CLSM, OLS4000, Olympus Co., Japan) pictures to quantitatively analyze the exfoliation corrosion resistance. The apparatus for exfoliation corrosion test is displayed in Figure 2. The immersion time for exfoliation corrosion test is 48 h and the size of the corrosion patch was around ϕ10 mm.

The electrochemical impedance spectroscopy (EIS) and potentiodynamic polarization tests are always be used to estimate local corrosion of aluminum alloys (IGC and exfoliation corrosion) [20,29]. An Reference 600 electrochemical workstation (America Gamry Electrochemical Instruments) and auxiliary equipment with a three-electrode system was used to analyze the electrochemical response to the specimens. The three-electrode system was consists of an Ag/AgCl electrode as a reference electrode, the platinum sheet as an anauxiliary electrode, and the Al-Zn-Mg alloy as a working electrode. EIS tests were carried out at open circuit potential (OCP) with a frequency range of 10 kHz to 0.1 Hz, and the sinusoidal perturbation of 10 mV RMS (root mean square) was used for EIS experiment. The analysis of EIS measurements results was performed by Zview software (3.0a, Solartron Metrology Co., West Sussex, United Kingdom). The potentiodynamic polarization experiment was carried out immediately after EIS test. The scan range is ±200 mV (relative to OCP) with a scan speed of 1 mVs^−1^. And the corresponding electrochemical parameters of corrosion current density (*i*_corr_) and corrosion potential (*E*_corr_) were got through Tafel-type fit of the polarization curves. In order to ensure the accuracy of test results, all electrochemical measurements were repeated three times or more. All electrochemical measurements were performed in the solution containing 1.0 M NaCl at room temperature and the testing size was around 0.28 cm^2^. 

The fracture surface morphology of heat-treated samples was observed by Zeiss Supra55 SEM equipment after tensile test. The samples suffered to different thermal cycles were prepared for Transmission electron microscope (TEM) observation. Thin foils for TEM were manufactured through twin-jet electropolishing with the mixture solution of HNO_3_:ethanol = 1:4 cooled to −25 °C at 15 V. TEM examination was performed at 200 kV on a Tecnai G^2^20 S-Twin (FEI Co., Portland, United States) apparatus. The change of elemental content in intergranular precipitates, matrix precipitates and precipitate-free zone was obtained through the energy dispersive spectroscopy (EDS, IE250X-Max50, Oxford Instruments, Oxford, United Kingdom) attached to TEM. For the accuracy EDS analysis, each data point was the arithmetic mean of at least 10 measured locations acquired at three different grain boundaries. 

## 3. Results

### 3.1. Tensile Strength

**Figure 3:**
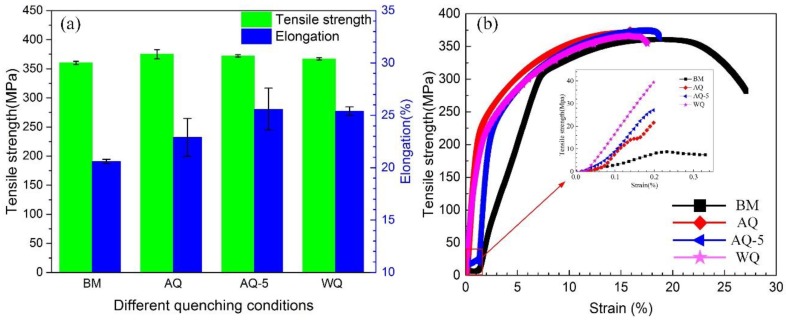
Tensile strength and elongation (**a**) and typical tensile stress-strain curves of heat-treated samples with different quenching conditions (**b**).

**Figure 4:**
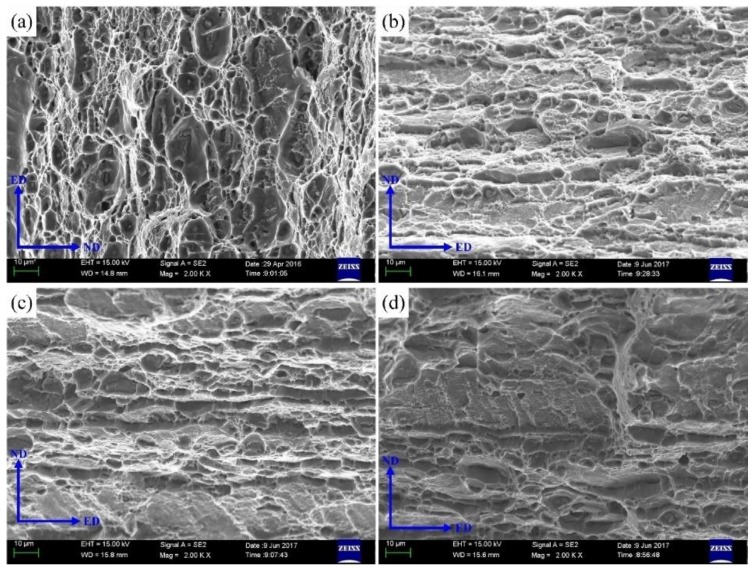
The fracture morphology of samples with different heat treatment process (**a**) BM, (**b**) AQ, (**c**)AQ-5 and (**d**)WQ (ED: extruding direction; ND: normal direction).

Figure 3 shows the evolution of mechanical properties of samples after NIHT with different quenching conditions. The tensile strength of samples of BM, AQ, AQ-5 and WQ is 360 MPa, 375 MPa, 372 MPa, and 367 MPa, respectively. The result shows that the tensile strength of the Al-Zn-Mg alloy has no obvious change after different quenching processes comparing to that of BM. The elongation of heat-treated samples AQ, AQ-5 and WQ are 23.0%, 25.6%, 25.4%, respectively, compared with that of BM (20.6%). A large number of Fine populated dimples and some cleavage steps can be observed in Figure 4. Some small dimples exist on the surface of the cleavage steps, indicating that the fracture mechanism of the specimen is ductile fracture. It is concluded that NIHT with different quenching conditions do not deteriorate the mechanical properties of Al-Zn-Mg alloy. The reason mainly related to the transformation of precipitates caused by different thermal cycles.

### 3.2. Corrosion Behavior

#### 3.2.1. Intergranular Corrosion

**Figure 5:**
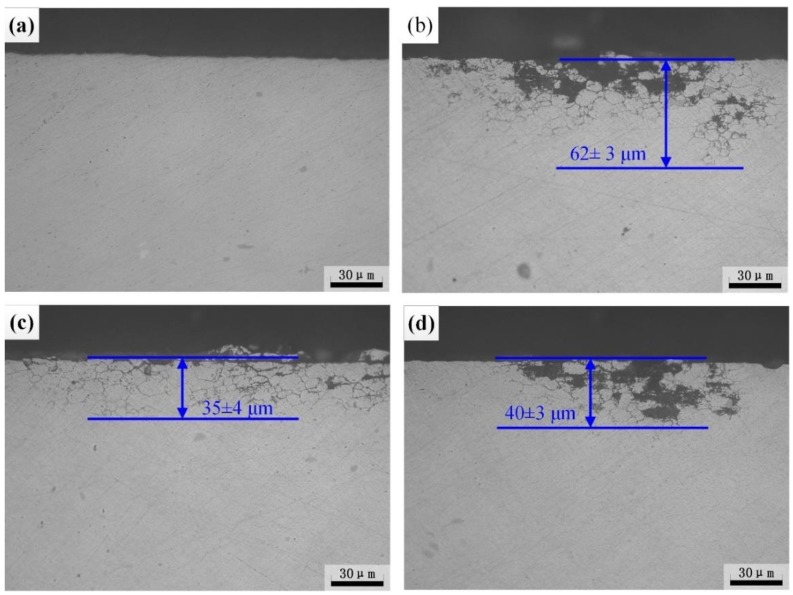
Metallographic images of BM (**a**) and heat-treated specimens with AQ (**b**), AQ-5 (**c**), and WQ (**d**) after IGC test.

Heat treatable Al-Zn-Mg alloy with different quenching technology will result in the evolution of precipitates and then affect the corrosion behavior [3,30,31,32]. The IGC morphology of heat-treated specimens is displayed in Figure 5. After different heat treatment processes, the IGC depth of the specimen AQ is 62 μm, which is most susceptible to IGC. However, the sample AQ-5 displays the highest resistance to IGC with the corrosion depth of 35 μm, as displayed in Figure 5b,c, respectively. The intergranular corrosion depth of sample WQ is 40 μm. For the BM, no obvious IGC characteristics appeared. It is concluded that the susceptibility to IGC of Al-Zn-Mg alloy increases after NIHT with different quenching processes.

#### 3.2.2. Exfoliation Corrosion

**Figure 6:**
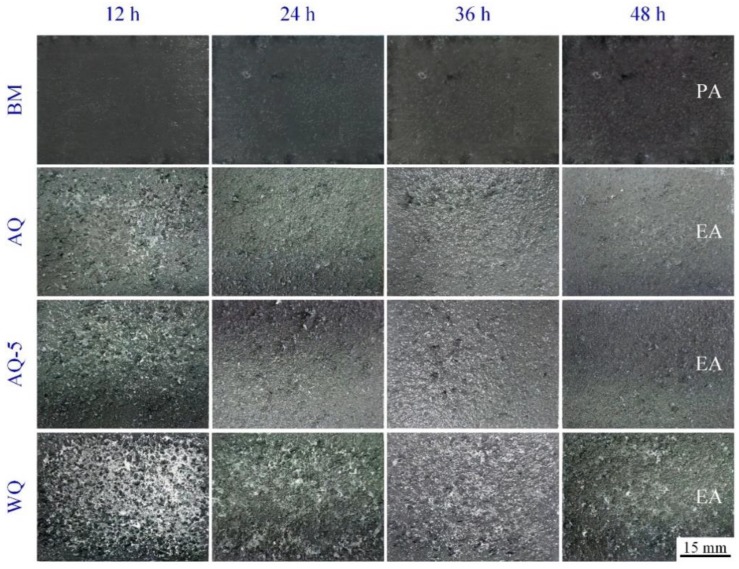
The evolution of exfoliation corrosion morphology of specimens with different quenching process.

**Figure 7:**
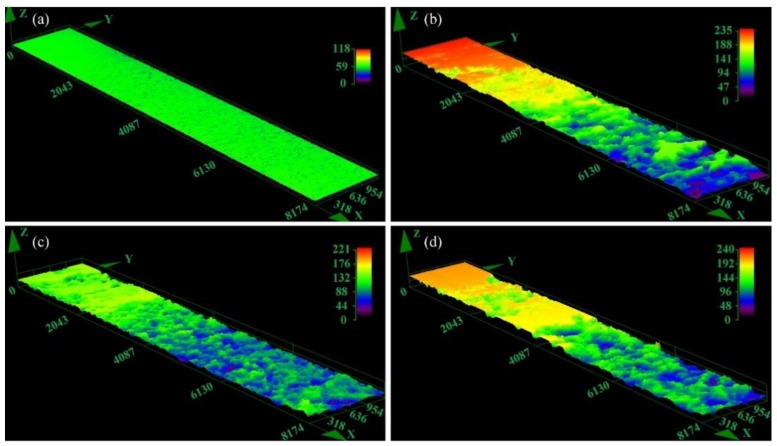
Three-dimensional optical micrographs without corrosion products after EXCO test: (**a**) BM, (**b**) AQ, (**c**) AQ-5, (**d**) WQ (Unit: μm ).

**Figure 8:**
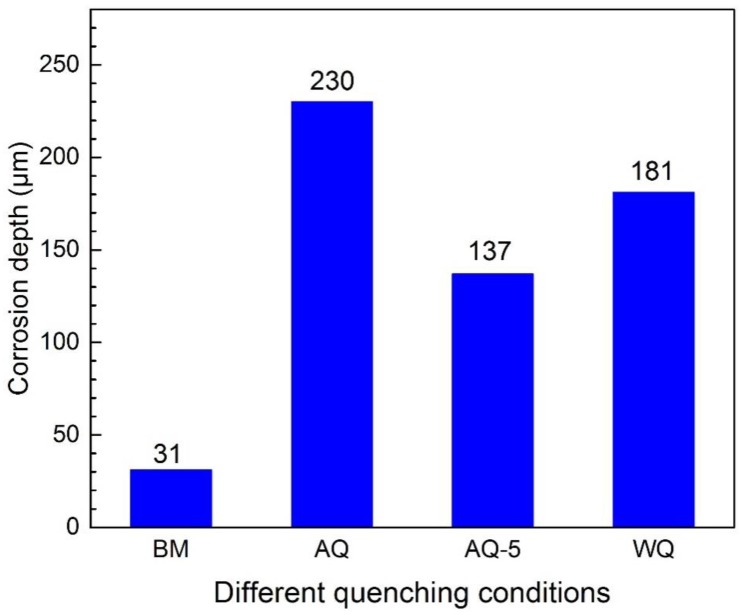
Corrosion depth without corrosion products after EXCO test of specimens with different quenching conditions.

The change of exfoliation corrosion feature of the specimens with different immersion time is shown in Figure 6. The exfoliation corrosion morphology of all heat-treated samples has no significant change by visual observation with the increase of immersion corrosion time. All exfoliation corrosion rating for heat-treated samples are assessed to be grade EA after immersion for 48 h. For the sample BM, the rank of exfoliation corrosion is assessed to be PA. Exfoliation corrosion is strongly affected by IGC. Additionally, the mechanical stresses, described as "wedging forces", also have obvious effect to corrosion failure [33]. Generally speaking, the ranking of exfoliation corrosion is estimated according to visual observation, which is both subjective and qualitative, so it is difficult to compare small variations between materials or different heat treatment conditions [6]. For instance, as displayed in Figure 6, the exfoliation rating may be the same for heat-treated specimens with different quenching conditions, however the depth of exfoliation corrosion may be different. The specimens were immersed in the mixed solution CrO_3_ 20 g/L + H_3_PO_4_ 50 mL/L to remove the corrosion products after exfoliation corrosion test. The three-dimensional morphology of specimens are obtained by using CLSM to evaluate the susceptibility to exfoliation corrosion quantitatively, as shown in Figure 7. Exfoliation corrosion belongs to local corrosion, consequently the maximum penetration depth of exfoliation corrosion of the samples was obtained by additional analysis software attached to CLSM facility and the corrosion depth is estimated by using the non-corrosive area as the datum plane, as shown in Figure 8. It is obvious that the BM shows better resistance to exfoliation corrosion with flat corrosion morphology, however, the morphology of the samples after NIHT with different quenching conditions displays typical exfoliation corrosion feature just like hilly terrain characteristics. The exfoliation corrosion feature of samples AQ-5 and WQ is relatively homogeneous, as shown in Figure 7c,d, respectively. However, the local exfoliation corrosion of specimen AQ is obvious, as shown in Figure 7b. The sample AQ is most susceptible to exfoliation corrosion with the maximum corrosion depth of around 230 μm, as illustrated in Figure 8a. The higher corrosion depth represents lower corrosion resistance. Consequently, the corrosion resistance of samples based on exfoliation corrosion can be ranked in the following order: BM> AQ-5>WQ>AQ, and the corresponding corrosion resistance variation trend is the same as the result obtained through the intergranular corrosion depth in Figure 5.

#### 3.2.3. Electrochemical Test

**Figure 9:**
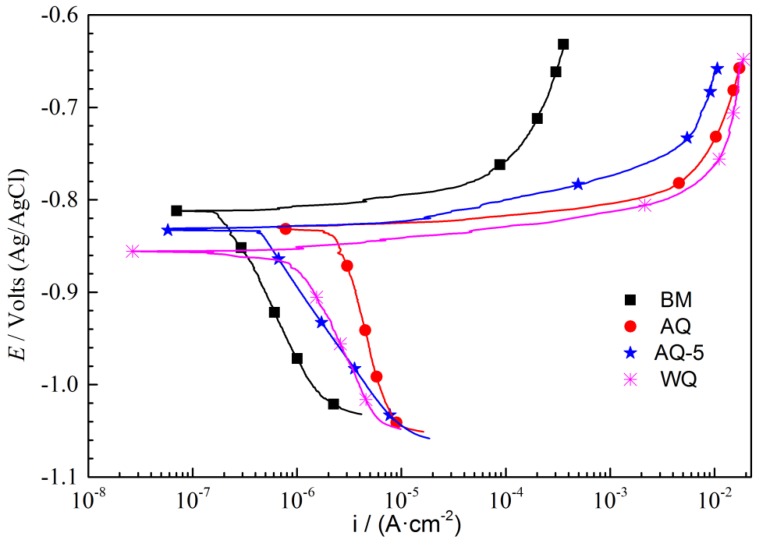
Polarization curves of samples with different quenching conditions. The scanning range is −200 mV to +200 mV (relative to open circuit potential), the test solution is 1.0 M NaCl, and the scan rate is 1 mV s^−1^.

The polarization curves of BM and heat-treated samples with different quenching conditions are shown in Figure 9. The relevant parameters are acquired through Tafel extrapolation (in Table 3). The corrosion current density (*i*_corr_) of BM and the samples after NIHT with different quenching procedures are different and ranks in the following sequence: AQ>WQ>AQ-5>BM. Generally speaking, the larger corrosion current density will give rise to larger corrosion rate. The change trend evaluated with corrosion current density is consistent well with the results of IGC test and exfoliation corrosion experiment, and proves that the selected data from dynamic potential polarization curves is appropriate to estimate the corrosion current density (*i*_corr_), although the fitting data could be subjective. The anodic current densities are gradually heading towards a plateau, which demonstrates that the pitting growth is becoming limited by a mass transport procedure. This behavior is likely associated with the appearance of a salt film within it [34].

**Figure 10:**
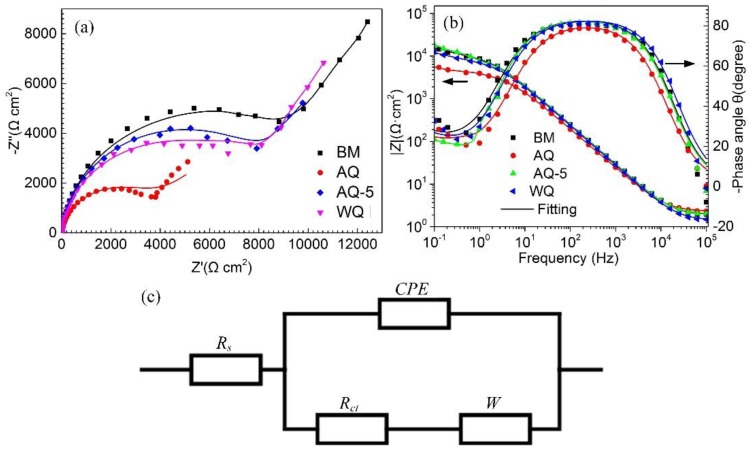
Effect of quenching condition on the impedance spectra of samples: (**a**) Nyquist plots, (**b**) Bode plots and (**c**) equivalent circuit used to fit the behavior of samples immersed in the solution. *R*_s_ represents solution resistance used in the test, CPE is constant phase angle element, *R*_ct_ and *W* is charge transfer resistance and Warburg impedance, respectively. The scattered symbols represent the experimental data and the solid lines represent the fitting results.

The EIS tests were carried out to study the electrochemical response of BM and the samples with different quenching processes. The test results are presented in the type of Nyquist and Bode-phase images, as shown in Figure 10. In the Nyquist image, a capacitive reactance arc forms in high-frequency area and a slash with a tilt angle appears in the low-frequency locations (in Figure 10a). This phenomenon reveals the existence of diffusion process known as Warburg impedance [35,36]. The transformation of EIS curve is mainly attributed to the change of the kinetics of corrosion procedure, possibly from charge transfer control to diffusion control. Additionally, the maxima of phase angle curves are lower than 90^o^, as shown in Bode plots of Figure 10b, which reveals the existence of a variance of ideal-capacitance with relative to the surface of specimens. The equivalent circuit in Figure 10c is applied to deal with the EIS data and the relevant electrochemical parameters are obtained through Zsimpwin software, as listed in Table 4. *R*_s_ means the solution resistance; *R*_ct_ and *W* represent charge transfer and Warburg impedance, respectively. Since there is no pure capacitance in actual electrochemical interface, a constant phase element (CPE) is usually applied to acquire a more accurate fitting to the electrochemical data. Liu et al. pointed out that the total resistance *R*_ct_ is inversely proportional to the corrosion rate [37]. The *R*_ct_ is always used to evaluate the susceptibility to corrosion, and the higher *R*_ct_ value represents a lower corrosion current density and thus a better corrosion resistance [20], as listed in Table 4. Consequently, the corrosion resistance of BM and heat-treated samples ranks in the following order: BM>AQ-5>WQ>AQ. Additionally, the deterioration of corrosion resistance can also be assessed by using *Y* values, and the corresponding variation trend of corrosion resistance is consistent with the laws evaluated through the *R*_ct_ values [37].From Table 4, it can be seen that all the values of CPE power are near 0.90, which reflects a higher interface homogeneity as previously studied by Chang et al. [38]. The change trend of corrosion resistance of BM and heat-treated samples with different quenching processes obtained through the EIS results is consistent well with the immersion experiments. 

### 3.3. Microstructure

#### 3.3.1. GBPs Structure and Microchemistry

**Figure 11:**
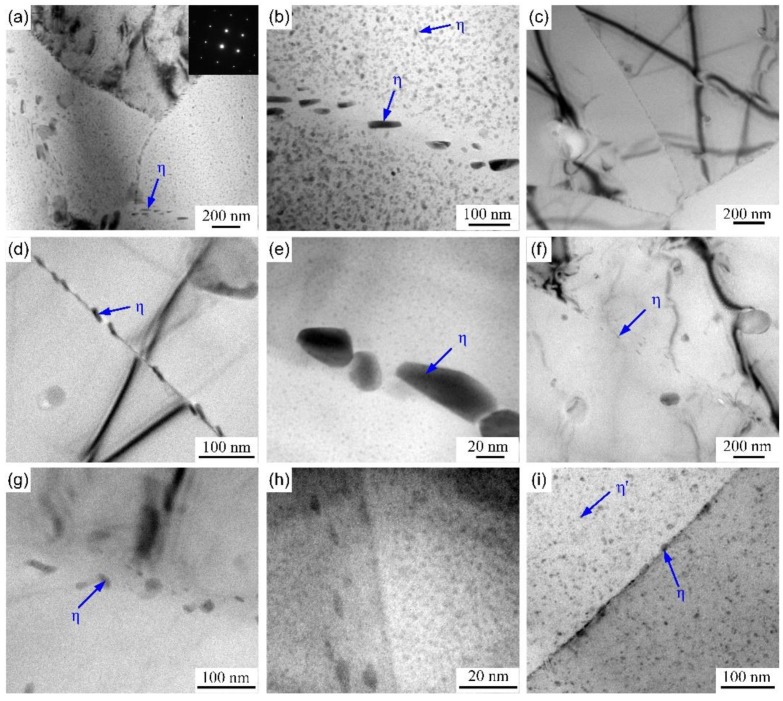
TEM observations for distribution of precipitates in (**a**,**b**) BM and heat-treated specimens with (**c**–**e**) AQ, (**f**–**h**) AQ-5, (**i**) WQ.

For 7000 series alloys, the widely recognized evolution of precipitation process is recommended as follows: supersaturated solid solution (α-sss)→Guinier-Preston zones (GP zones, MgZn)→η′ phase (MgZn_2_)→η phase (MgZn_2_) [39]. The Al-Zn-Mg alloy belongs to heat-treatable aluminum alloy and the precipitates will change due to different thermal cycles [1,16,40]. Figure 11 shows the precipitates evolution of heat-treated Al-Zn-Mg alloy with different thermal cycles. The microstructure of BM is composed of high densities η′ and η precipitates in matrix, discontinuous precipitates at grain boundaries and precipitate-free zones near the grain boundaries, as shown in Figure 11a,b. For sample AQ, almost all precipitates in matrix (η′ and η phases) dissolve judging from Figure 11c,d. However, high densities GP zones appear in higher magnification image in Figure 11e, which most likely attribute to the natural ageing after NIHT. Additionally, the precipitates at grain boundary of sample AQ suffered dissolution and re-precipitation, and then become continuous, which is harmful to the corrosion resistance of Al-Zn-Mg alloy [6,20,23]. The matrix precipitates of sample AQ-5 have similar evolution process except for precipitates at grain boundary, as seen in Figure 11f–h. The grain boundary precipitates of sample AQ-5 show discontinuous morphology, which is benefited to decrease the susceptibility to corrosion. There are still some matrix and grain boundary precipitates in sample WQ, which can be ascribed to different quenching conditions during NIHT process, as shown in Figure 11i. 

**Figure 12:**
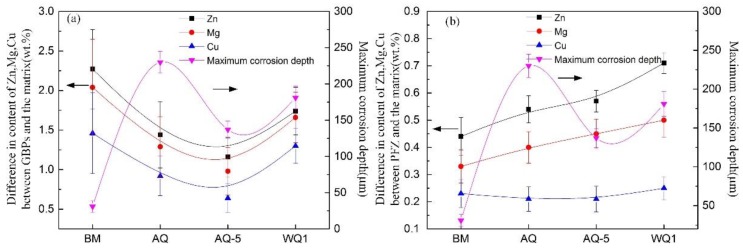
Variation of difference in Zn, Mg, and Cu contents (**a**) between grain boundary precipitates (GBPs) and the matrix, and (**b**) between precipitate-free zones and the matrix with different quenching conditions.

The microchemistry was also analyzed by measuring the chemical compositions of GBPs, PFZs and matrix. In Song’s [23] and Liu’s [20] work, the variation of Zn, Mg, Cu of grain boundary precipitates and precipitate-free zones near grain boundaries with different quenching rates was examined, but they neglected the change of elements in matrix. It was mainly related to the heat treatment process in their papers. The relevant heat treatment procedure can be summarized as follows, solution heat treatment + quenching (with different quenching rate) + artificial ageing. In the last step of artificial ageing, lots of precipitates in matrix formed and the corresponding corrosion potential of matrix increased due to the decrease of Zn content. The relevant investigations have proven that the corrosion potential of GBPs and PFZs are higher than that of matrix [6,20,23,30]. Therefore, only the variation of element in grain boundary precipitates and precipitate-free zones was considered. The microstructure evolution in matrix of heat-treated specimens, namely from η′ or η precipitates to GP zones formed in natural ageing, will lead to the increase of Zn content in matrix [41,42], and then lead to the decrease of potential in matrix. The element content in grain boundary precipitates and precipitate-free zones will be affected by the diffusion from matrix to grain boundaries and the evolution of precipitates at grain boundary, the change has complex effect on the potential of grain boundary precipitates and precipitate-free zones. Then these factors affect the corrosion resistance of heat-treated specimens with different quenching conditions. The relevant investigations have pointed out that the variation of corrosion resistance for 7000 series alloy is primarily related to two factors [6,20,23,43]: (I) the area fraction of grain boundary precipitates and the width of precipitate-free zones; (II) the corresponding potential difference between the aluminum matrix and grain boundary precipitates, precipitate-free zones. Consequently, the Zn, Mg, Cu contents differences between the grain boundaries and matrix, and the precipitate-free zones and matrix are counted, as shown in Figure 12. From Figure 12a, the difference of Zn between GBPs and matrix is larger than that of Mg and Cu. In addition, the difference of Zn, Mg and Cu between precipitates at grain boundary and matrix displays same variation trend with different quenching conditions, namely gradually decreased from the BM to specimens AQ-5, and then increased. Figure 12b shows that the difference of Zn and Mg between precipitate-free zones and matrix increases from BM to specimen WQ, however, the difference of Cu content remains almost unchanged. 

#### 3.3.2. Differential Scanning Calorimeter (DSC) Analysis

**Figure 13:**
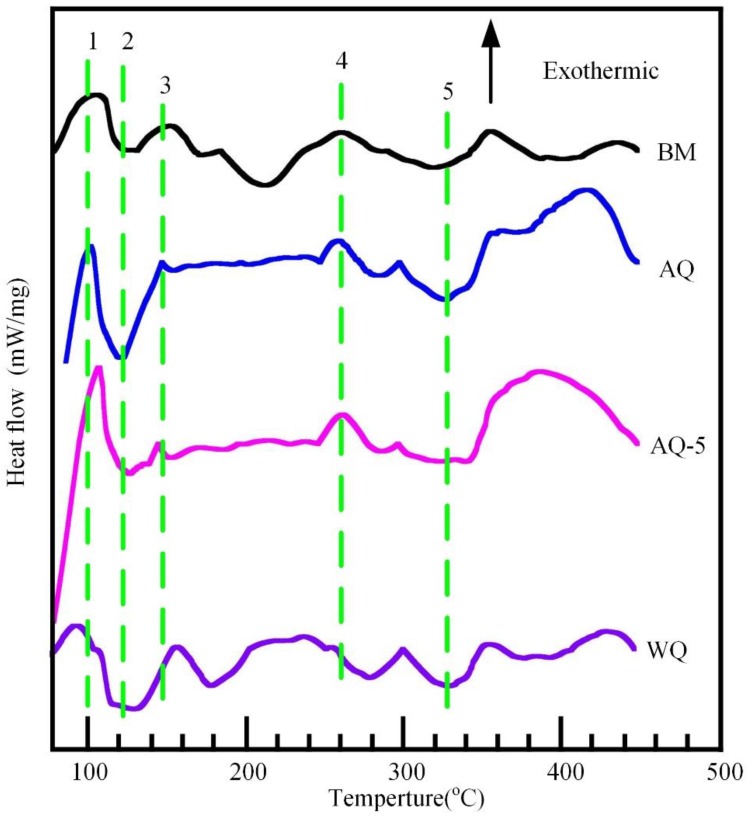
DSC curves of specimens with different quenching conditions. The curves have been offset for clarity.

DSC experiment is always be used in conjunction with TEM investigations to characterize the microstructural evolution, as shown in Figure 13. The exact locations of all peaks are related to the alloy composition, heating rate and the initial state of heat treatment prior to DSC test [44,45]. For the sample BM, an exothermic reaction (peak 1) can be obtained with the temperature range from 75 °C to 110 °C, demonstrating the formation of GP zones [44,46]. The endothermic peak 2 covering 110 °C–130 °C range is most likely associated with the dissolution of GP zones. Subsequently, a slight exothermic reaction (peak 3) occurred in 130 °C–170 °C span corresponding to the precipitation of η′ phases. The exothermic reaction (peak 4) is subsequently acquired with the temperature range of 210 °C–280 °C, which indicates the formation of η phases. The dissolution of η′ and η phases occur when the temperature increase, resulting in the formation of endothermic reaction (peak 5) in 280 °C–350 °C span. The exothermic and endothermic reactions are related to the appearance and dissolution of S and T precipitates when the temperature is higher than 350 °C [44,47]. The variation trend of DSC for sample WQ is similar with that of specimen BM. However, the intensity of exothermic peak 3 for specimen WQ is obvious compared with the sample BM, which is most likely attributed to the difference of initial temper of samples. For samples AQ and AQ-5, the very wide temperature range of endothermic peak 2 (~100 °C–190 °C) suggests that lots of GP zones dissolved during DSC test. It means that lots of GP zones appeared during natural ageing process after NIHT. Additionally, it is likely that some reactions were overlapped under the endothermic peak 2, such as peak 3 [44]. The DSC analysis indicates the difference of precipitates in BM and heat-treated specimens. 

## 4. Discussion

In Figure 3, it can be seen that quenching process has no obvious effect on the tensile strength of specimens. However, the results of IGC and exfoliation corrosion test suggest that the corrosion susceptibility of Al-Zn-Mg alloy changes obviously after NIHT with different quenching conditions. The relevant investigations show that the tensile strength of Al-Zn-Mg (Cu) alloys is mainly associated with the precipitates of GP zones and η’ phase [48,49,50,51], while there is currently no agreement about their effect on the mechanical properties of Al-Zn-Mg (Cu) alloys [50,51,52,53,54]. Actually, it is difficult to confirm this argument experimentally due to the temperature ranges of the formation of GP zones and η′ precipitates are overlapping. Additionally, they are small in size. The evolution of tensile strength of the samples after NIHT with different quenching condition is likely caused by change of the volume fractions of GP zones and η′ precipitates, that is to say, the formation of GP zones compensate the strength loss by the dissolution of original precipitates. Additionally, there are no obvious precipitate-free zones after NIHT with different quenching conditions, which contribute to the improvement of ductility [55].

Based on the electrochemical and accelerated immersion test results, the NIHT deteriorated the corrosion resistance of Al-Zn-Mg alloy. The corrosion resistance of heat-treated specimens with different quenching conditions can be ranked in the following order: AQ-5> WQ > AQ. The local corrosion (IGC and exfoliation corrosion) is mainly related to microstructure and microchemistry near grain boundary [20,23]. It can be seen that the quenching condition has complex influence on the evolution of microstructure and microchemistry according to the present work. The relevant investigations prove that the corrosion resistance of Al-Zn-Mg (Cu) aluminum alloys is associated to the distribution of grain boundary precipitates, such as the continuity of η phase and the volume fraction of η precipitates at grain boundaries [6,23,30,56]. It was demonstrated that larger and more dispersed grain boundary precipitates can increase the corrosion resistance and higher coverage ratio of grain boundary precipitates contributes to higher corrosion propagation along grain boundaries [6,23,30,56]. However, the evolution of corrosion resistance after NIHT with different quenching conditions can’t be interpreted directly, judging from the distribution and morphology of grain boundary precipitates, as shown in Figure 11. Consequently, the microchemistry near grain boundaries should be considered. 

The grain boundary precipitate η and precipitate-free zones have anodic feature compared with the matrix. The grain boundary precipitates tends to be corroded first, then the precipitates free zones will be attacked [6,19,20,23,57]. Actually, the corrosion susceptibility of Al-Zn-Mg (Cu) alloys is mainly related to the electrochemical potential difference between the matrix and the grain boundary precipitates, precipitate-free zones, respectively [20,23]. The evolution of electrochemical potential difference is presented by the variation of Zn, Mg and Cu contents, which has complex influence on the electrochemical potential of the grain boundary precipitates, precipitate-free zones and matrix. Liu et al. [20] pointed out that Zn and Mg contents increase more obviously than that of Cu content with the reduction of quenching rate. Then the negative influence of Zn and Mg contents exceeds the positive effect of Cu content, leading to larger electrochemical potential differences between grain boundary precipitates and adjacent precipitate-free zones, consequently the precipitates at grain boundary are corroded more easily in corrosion medium. In this paper, for the specimen of BM in Figure 12a, the difference in Zn, Mg and Cu contents between the precipitates at grain boundary and matrix is higher than those of other specimens. Nevertheless, the corrosion resistance of BM is better than that of other samples. It seems that this phenomenon occurs because the positive effect of difference in Cu content exceeds the negative effect of difference in Zn and Mg contents between the grain boundary precipitates and matrix, according to Ref. [20]. However, for the specimen AQ-5, the difference in Zn, Mg and Cu contents between the grain boundary precipitate and matrix decreases and the corrosion resistance decreases. Consequently, the evolution of corrosion resistance after NIHT with different quenching conditions can’t be explained simply by the difference of positive effect of Cu or negative influence of Zn, Mg on the electrochemical potential. The morphology of grain boundary precipitates and the electrochemical potential difference between the precipitate-free zones and matrix should be considered. 

The investigations about the continuity [30,32,56], the coverage ratio of grain boundary precipitates [6] and width of precipitate-free zones [23] on the effect of corrosion resistance seems imply that the corrosion rate of precipitate at grain boundary and precipitate-free zone is different [20]. 

As for the sample BM, the formation of η phase at grain boundary during artificial ageing can result in the occurrence of solute-depleted zone, i.e. the precipitate-free zone. Consequently, the difference in Zn, Mg and Cu contents between grain boundary precipitates and matrix is higher, while the difference of Zn, Mg and Cu contents between the precipitate-free zone and matrix is lower. The specimen of BM shows better corrosion resistance judging from the immersion test results in Figure 5, Figure 6 and Figure 7. One reason is likely because of the lower volume fraction of grain boundary precipitates although the difference of electrochemical potential between the grain boundary precipitates and matrix [6]; another reason is associated with lower difference in Zn and Mg contents between the precipitate-free zones and matrix, as shown in Figure 12b, which means lower difference in electrochemical potential. For the sample AQ, the grain boundary precipitates suffer from dissolution and re-precipitation process after NIHT with air quenching. The precipitates at grain boundaries of sample AQ were not subjected to long time artificial ageing compared to the BM. Therefore, the difference of Zn, Mg and Cu contents between the grain boundary precipitates and matrix decreases, while the corresponding difference between the precipitate-free zones and matrix increases, as shown in Figure 12. The distribution of grain boundary precipitates of sample AQ is more continuous, which is the main effect factor on the corrosion resistance and then promotes to the corrosion propagation along grain boundaries, as shown in Figure 11e, resulting the higher corrosion susceptibility. The grain boundary precipitates for specimen AQ-5 also subject to dissolution and re-precipitation process, while the difference in Zn, Mg and Cu contents between the grain boundary precipitates and matrix decreases due to shorter duration in high temperatures. And the corresponding difference in Zn, Mg and Cu contents between the precipitate-free zones and matrix increases. Additionally, the precipitates at grain boundary become more discontinuous. These combination factors contribute to get better corrosion resistance for sample AQ-5. For the sample WQ, the precipitate can dissolve into the matrix when the dimension of precipitates is smaller than that of critical radius of dissolution of precipitates [16], and the larger precipitates may be subjected to partly dissolution as shown in Figure 11i. However, there is no enough time to diffuse to a status of uniformity for the vast solute elements in matrix, so they still exist in the surrounding of residual precipitates. Then the electrochemical difference between matrix and grain boundary precipitates, precipitate-free zones increases respectively. Consequently, the susceptibility to corrosion increases compared with the specimen AQ-5. Additionally, the residual stress is also different between the samples AQ-5 and WQ, it is maybe another reason for the difference of local corrosion.

## 5. Conclusions

(1) The corrosion resistance of Al-Zn-Mg alloy decreases after non-isothermal heat treatment, and the sample with air quenching process has the lowest corrosion resistance. While, the specimens can get better corrosion resistance when suffered to 5 min air cooling and then water quenching process. 

(2) The tensile strength of base metal and samples after non-isothermal heat treatment with different quenching conditions have no significantly change, however the elongation of heat-treated samples increases, compared with that of base metal.

(3) The change of mechanical properties and corrosion susceptibility of heat-treated specimen is caused by the modification of distribution of grain boundary precipitates and the microchemistry of precipitates at grain boundary, precipitate-free zones and matrix. 

## Figures and Tables

**Table 1:** Chemical composition of Al-Zn-Mg alloy extrusion plate (wt.%).

Zn	Mg	Mn	Cr	Zr	Fe	Cu	Ti	Si	V	Al
4.480	1.547	0.294	0.232	0.180	0.125	0.113	0.054	0.050	0.014	Bal.

**Table 2:** Quenching conditions and acronyms of the samples.

Samples	Quenching Process
BM	Base metal
AQ	Air quenching
AQ-5	5 min air quenching and then water quenching
WQ	Water quenching

**Table 3:** Heating parameters and electrochemical parameters obtained from polarization plots.

Sample No.	*E*_corr_ (mV) vs(Ag/AgCl)	*i*_corr_ × 10^-7^ (A·cm^−2^)	*β_a_* (mV·dec^−1^)	*β_c_* (mV·dec^−1^)
BM	−815 ± 2	2.0 ± 0.3	16 ± 5.6	−242 ± 3.7
AQ	−829 ± 2	13.9 ± 1.6	11.5 ± 2.6	−39 ± 2.8
AQ-5	−832 ± 4	4.2 ± 0.3	19.8 ± 2.4	97 ± 4.5
WQ	−860 ± 3	5.6 ± 0.5	20 ± 3.3	−230 ± 6.4

**Table 4:** Electrochemical parameters obtained from EIS analysis.

Sample No.	*R*_s_ (Ω·cm^2^)	CPE		*R*_ct_ (kΩ·cm^2^)	*Y_w_* (10^−4^·Ω^−1^·cm^−2^·s^−0.5^)
		*Y_0_* (10^−6^·Ω^−1^·cm^−2^·s^−n^)	*n* (0 < n < 1)		
BM	1.9	9.77 ± 0.39	0.93	8.65 ± 0.49	1.39 ± 0.04
AQ	2.3	14.40 ± 0.39	0.91	3.52 ± 0.10	3.17 ± 0.19
AQ-5	1.9	10.11 ± 0.28	0.93	8.32 ± 0.36	1.68 ± 0.04
WQ	1.4	10.30 ± 0.33	0.93	6.73 ± 0.27	1.82 ± 0.06
